# Associations between adolescent adversity and young adult depression symptoms and allostatic load in Mexican-origin individuals

**DOI:** 10.1016/j.psyneuen.2026.107832

**Published:** 2026-03-08

**Authors:** Madeleine R. Frazier, Amanda E. Guyer, Paul D. Hastings, Richard W. Robins, Johnna R. Swartz

**Affiliations:** a Department of Human Ecology, UC Davis, CA, USA; b Center for Mind and Brain, UC Davis, CA, USA; c Department of Psychology, UC Davis, CA, USA

**Keywords:** Perceived discrimination, Economic hardship, Adversity, Allostatic load, Depression, Mexican origin

## Abstract

Childhood adversity increases risk for depression and higher allostatic load (AL), defined as long-term wear and tear on the body, and adolescence is an especially sensitive time for adversity to be biologically embedded. Individuals of Mexican origin may face multiple forms of adversities relating to their intersectional identities of ethnicity and class, including ethnic discrimination and economic hardship, the experience of which may further depend on sex. However, relatively little research has simultaneously assessed associations between these two types of adolescent adversity and outcomes of both young adult depression and AL longitudinally in Mexicanorigin individuals. This preregistered study addressed these gaps in a longitudinal study of 208 Mexicanorigin participants. Ethnic discrimination and economic hardship were assessed annually from ages 10–19 and AL and depression symptoms were assessed at age 26. Sex differences were also tested. Perceived discrimination in adolescence significantly predicted higher levels of depression at age 26 (β =.09, *p* = .045, R^2^ =.03, ΔR^2^ = 0.005, 95% CI [.002,.181]). In addition, age 26 depression levels moderated the association between adolescent economic hardship and age 26 AL (β = −.17, *p* = .03, R^2^ =.12, ΔR^2^ =.028, 95% CI [−.33, −.02]). Participants exhibited higher AL with high levels of economic hardship but low levels of depression, as well as with low levels of economic hardship and high depression, suggesting that both psychological resilience to economic hardship and the association between mental health and AL may depend on context. Findings indicate intersectional effects of different types of adversity during adolescence on mental and physical health in young adulthood within Latino individuals.

## 1. Introduction

Adolescence is a sensitive developmental period when trajectories of vulnerability and resilience to stress-related mental and physical health problems are malleable, given rapid neurobiological changes during this time (Mancini et al., 2023). Racially and ethnically minoritized youth are more often exposed to manifold adversities compared to majority youth and may therefore be at a higher risk for stress-related disorders in adulthood such as Major Depressive Disorder (MDD) and chronic physical problems (Archambault et al., 2017; Hastings et al., 2022; Marks et al., 2020). According to allostatic load (AL) theory (Seeman et al., 2001), chronic stress causes stress physiology dysfunction over time in which the body’s capacity to efficiently and flexibly respond to everyday stressors becomes limited. This results in dysregulation of multiple biological systems, including the hypothalamic-pituitary-adrenal (HPA) axis, immune, cardiovascular, and metabolic systems. Existing evidence shows that adults with higher AL are also more likely to exhibit MDD (Honkalampi et al., 2021), suggesting a link between AL and MDD, although these have often not been examined together within one study in younger participants.

An intersectionality framework lends nuance to models of stress accumulation by accounting for the ways in which multiple elements of social inequality related to one’s intersectional identities impact health concurrently (Hankivsky et al., 2017). Thus, it is important to study the long-term health impacts of multiple systems of inequity for minority youths, especially with longitudinal study designs, which are lacking in the literature (Harari and Lee, 2021). The present study uses data from the California Families Project (CFP), which has followed participants longitudinally from age 10–26, to examine associations between two forms of adversity related to inequity (i.e., adolescent ethnic discrimination and economic hardship) and young adult physical and mental health outcomes.

Prior research suggests that perceived discrimination elevates risk for MDD and higher AL. Among Latino^1^ individuals, empirical studies and reviews have consistently shown that discrimination is associated with worse mental health outcomes, including clinical depression, suicidal ideation, and psychological distress (Araújo and Borrell, 2006; Hwang and Goto, 2008). When examining physical health outcomes across various races, chronic experiences of discrimination have been linked to altered cortisol diurnal rhythms and higher AL in adulthood (Adam et al., 2015; Brody et al., 2014; Cuevas et al., 2019). However, no research to our knowledge has examined the longitudinal association between adolescent discrimination and adult AL in a Latino sample.

In a separate line of research, studies have shown that low income and socioeconomic status, including childhood poverty, are associated with increased risk for MDD in adulthood (McLaughlin et al., 2011; Yali et al., 2022). When examining physical outcomes, research shows socioeconomic adversity is cross-sectionally linked to higher AL scores in youths of various racial and ethnic backgrounds (Gallo et al., 2019; Rainisch and Upchurch, 2013), and longitudinal studies have found that early childhood poverty is associated with increased young adult AL (Evans and De France, 2022; Evans and Kim, 2012).

An intersectionality framework also suggests that mental and physical health outcomes of adversity may differ for Latino women and men due to the cumulative stressors of holding multiple marginalized identities. Previous research has found that the prevalence of MDD is higher in women (e.g., Weissman et al., 1993) and sex differences have also been found in AL (e.g., Richardson et al., 2021) as well as for the association between depression and AL (Bey et al., 2018; however, see Cuevas et al., 2019). Despite these differences, little research to our knowledge has tested whether effects of adversity on MDD and AL in Latino individuals differs for men and women.

Furthermore, even positive psychosocial development in the face of adversity may place strain on the body over time because of long-term high-effort coping. For example, a longitudinal study of rural African American youths found that better self-control in childhood predicted fewer depressive symptoms yet higher AL in late adolescence, reflecting a tradeoff between mental well-being and physical health under conditions of contextual adversity termed ‘skin-deep’ resilience (Brody et al., 2013). Similarly, upward socioeconomic mobility and college completion were prospectively associated with fewer depressive symptoms but higher rates of metabolic syndrome in the Add Health study (Crosnoe and Thorpe, 2022; Gaydosh et al., 2018; Miller et al., 2020). However, Latino populations continue to be underrepresented in this work.

Collectively, the research reviewed above suggests that chronic inequity-related stressors increase risk for depression and AL. Theoretical frameworks suggest this occurs through chronic activation of the stress response due to repeated experiences of discrimination (in the case of perceived discrimination; Berger and Sarnyai, 2015) or due to family economic pressure and resulting disruptions in the parent-child relationship (in the case of adolescent economic hardship; Masarik and Conger, 2017). Chronic activation of the stress response, including the HPA axis and sympathetic-adrenal-medullary axis, in turn, can lead to long-term changes to the metabolic, immune, and cardiovascular systems influenced by these axes, such as hypertension, obesity, diabetes, atherosclerosis, or inflammatory disorders (Berger and Sarnyai, 2015; McEwen, 1998). For example, frequent or intense increases in blood pressure due to chronic activation of the stress response can damage blood vessels and lead to atherosclerosis (see McEwen, 1998 for an extended review of mechanisms). Considering intersectionality, this association may be complex for youths with several marginalized identities because they are more likely to experience chronic stressors from multiple sources. Although some studies have found that perceived discrimination and economic hardship increase risk for MDD and higher AL, other studies have documented a potential tradeoff effect in which psychological resilience (i.e., lower depression symptoms) in the context of adversity is associated with higher AL.

Therefore, the goal of the present study was to test these two competing models to clarify associations between adolescent adversity (specifically, perceived discrimination and economic hardship) and young adult depression and AL in a sample of Mexican-origin^2^ individuals. The first aim was to investigate whether elevated levels of economic hardship and ethnic discrimination would be associated with higher depression symptoms and higher AL at age 26. The second aim was to investigate whether depression and AL would be positively associated. The third aim was to test the ‘resilience is skin deep’ model by examining whether individuals who reported low levels of depression symptoms at age 26, despite high levels of adolescent adversity, had higher levels of AL. Finally, informed by an intersectional approach, we conducted exploratory analyses testing whether sex assigned at birth (hereafter sex) moderated these pathways.

## 2. Method

### 2.1. Study procedures and participants

This study was preregistered on Open Science Framework: https://osf.io/p4kub. Deviations to the preregistration are reported in Supplement Table S1. Participants were from the California Families Project (CFP), a longitudinal study of 674 Mexican-origin families in Northern California. The study began when children (50% female) were approximately 10 years old in 2006 and has followed them over time through age 26. Children were randomly drawn from school district rosters in Sacramento and Woodland, CA. To be eligible to participate, families had to self-identify as being of Mexican heritage, and the target child within a given family needed to be living with their biological mother. For two-parent families, the father needed to be the child’s biological father, and families with single mothers were eligible if there was no other adult residing in the household. Participants were interviewed by bilingual interviewers, most of whom were of Mexican heritage, in their homes in Spanish or English depending on their preference. Most families were bilingual, speaking both Spanish and English and speaking predominantly Spanish at home.

Child participants annually reported on perceptions of discrimination, and their mothers reported on household income and household size every year as well as economic security every other year. At approximately age 26, participants were assessed on a range of markers indicative of AL and symptoms of depression. This study used all available participants’ data on perceived discrimination and economic hardship experienced during adolescence (ages 10–19) as well as symptoms of depression and AL measures in young adulthood (age 26; see Table 1 for sample sizes available for these variables). Parents provided written consent for adolescent participants, adolescent participants provided assent, and when they were adults, consented independently. All participants were compensated monetarily for their participation and efforts in this study, which was approved by the Institutional Review Board (IRB) at the University of California, Davis.

### 2.2. Measures

#### 2.2.1. Economic hardship

Economic hardship during adolescence consisted of measures of income-to-needs ratio, assessed at every wave of data collection, and economic security, assessed at every other wave of data collection. Income-to-needs ratios were calculated for each wave (ages 10 – 19) based on mothers’ reports of total household income to the nearest $5000 increment and how many people were living in the household, following previous work with the CFP sample (Weissman et al., 2018). Economic security was assessed by asking participants’ mothers to report on economic stressors every other year using three scales (Conger et al., 2002, 1991). The Unmet Material Needs scale consisted of six items asking about whether families were able to meet their basic material needs (e.g., housing, food) rated from 1 (*not at all true*) to 4 (*very true*). The Financial Cutbacks scale consisted of 9 items asking about adjustments to financial need (1 = yes, 2 = no) during the past 3 months. The Can’t Make Ends Meet scale consisted of two items asking mothers how much money was left at the end of the month on a scale from 1 (*more than enough money*) to 4 (*not enough to make ends meet*) and whether they had difficulty paying their bills on a scale from 1 (*no difficulty at all*) to 4 (*a great deal of difficulty*). Items on the Can’t Make Ends Meet scale were reverse coded and then scores from the three questionnaires were summed to create a continuous variable of economic security. Internal consistency for each questionnaire at each wave is reported in Table S2.

Income-to-needs ratios and economic security scores were standardized by converting the variables to *Z*-scores. For overlapping waves of measurement (i.e., when adolescents were ages 10, 12, 14, 16, and 19), standardized scores for economic security and income-to-needs ratio were averaged to create a composite index of economic hardship. We then averaged the economic hardship composite across all available waves from age 10–19. This strategy allowed us to include participants missing data at one or more waves of measurement; however, we excluded participants missing data for both measures on more than half the waves (*n* = 66). The composite economic hardship measure showed excellent internal consistency, Cronbach’s α = .91 (Nunnaly and Bernstein, 1994). Finally, we multiplied composite scores by −1 so that higher scores corresponded to greater levels of economic hardship in order to aid in interpretation.

#### 2.2.2. Perceived discrimination

Perceived discrimination during adolescence was assessed annually using the Perceptions of Discrimination Scale, adapted for adolescents by Johnston and Delgado (2004) from measures of sexist events and racism in the workplace (Hughes and Dodge, 1997; Klonoff and Landrine, 1995; Schriber et al., 2018). For analyses, we used average scores from the Personal Experiences with Prejudice and Discrimination subscale that asked about adolescents’ personal experiences with discrimination from teachers, peers, neighbors, and strangers on a scale ranging from 1 (*almost never or never*) to 4 (*almost always or always*). To obtain a single measure of adolescent discrimination, we averaged mean scores from each year of measurement across all available waves from age 10–19, and we excluded those missing data on more than half the waves of measurement (*n* = 74). The composite measure of perceived discrimination showed moderate internal consistency, Cronbach’s α = .66 (Nunnaly and Bernstein, 1994). Internal consistency for each wave is reported in Table S2.

#### 2.2.3. Depression

We created a composite measure of depression symptoms at age 26 by combining two commonly used measures of depression: the Diagnostic Interview Schedule for DSM-5 (DIS-5; Compton and Cottier, 2004; Robins et al., 1981) and the Mini-Mood and Anxiety Symptom Questionnaire (MASQ; Lin et al., 2014; Watson et al., 1995). Participants were interviewed in person using the DIS-5 (n = 503). We used past year symptom counts from the Major Depressive Disorder (MDD) module of the DIS-5 (Cronbach’s α =.96), which could range from 0 to 9. From the MASQ (n = 507), we used average scores from the General Distress (n = 504; α =.93) and Anhedonic Depression (n = 505; α =.91) subscales, both rated on a 4-point scale from 1 (not at all) to 4 (very much), with the anhedonia scale reverse-scored so that higher scores indicated more anhedonia.

We created a composite variable by standardizing the MASQ General Distress subscale, the MASQ Anhedonic Depression subscale, and the DIS-5 Major Depression module symptom counts and then summing these three values. The composite measure showed acceptable internal consistency, α = .70 (Nunnaly and Bernstein, 1994). For any significant effects observed, we also conducted sensitivity analyses to examine whether results changed when individually analyzing each of the three measures of depression used in the composite score.

#### 2.2.4. Allostatic load

AL biomarkers were collected at age 26 during home visits. AL analyses included participants who provided data on eight total biomarkers described below (blood pressure, waist-to-hip ratio, body mass index, and five blood biomarkers; *n* = 217). Using an indexing approach established in prior research (Brody et al., 2013; Evans and Kim, 2012; Seeman et al., 2001), we first classified participants into risk quartiles for each AL marker relative to the sample. Then, we calculated a sum score for each participant representing the number of markers for which they were within the highest risk quartile. Because there are eight markers, this AL score has a possible range of 0–8. We also conducted sensitivity analyses with a subsample of participants who provided at least four out of eight biomarkers at the age 26 assessment (*n* = 288).

##### 2.2.4.1. Blood pressure.

Resting blood pressure was measured with an upper arm monitor (Omron Healthcare; https://omronhealthcare.com/blood-pressure/). Three readings were taken with two minutes between each reading; measurements for systolic (SBP) and diastolic (DBP) blood pressure were recorded. In line with prior research (Evans and Kim, 2012; Seeman et al., 2001), we used the average of the second and third readings as an index of blood pressure. The following cutoffs were used to flag implausible values: SBP < 60, SBP > 300, DBP < 30, DBP > 130. One participant was excluded for a DBP rating of 133.50.

##### 2.2.4.2. Waist to hip ratio.

Waist and hip circumference were measured with non-stretch dressmaker’s tape to the nearest 0.5 in. Waist to hip ratio was calculated by dividing the waist measurement by the hip measurement.

##### 2.2.4.3. Body mass index (BMI).

Weight and height were measured with a scale and tape measure, respectively. We used the following formula to calculate BMI in accordance with previous work (Beckie, 2012): BMI = weight (kilograms) / height (meters^2^).

##### 2.2.4.4. Blood biomarkers.

Microliter capillary whole blood samples were collected from participants’ arms using the Tasso+ kit blood lancet (Tasso Inc, Seattle, WA; https://www.tassoinc.com/tasso-plus-kit). Samples were shipped to an accredited laboratory, and the following markers were obtained: High-sensitivity C-reactive protein; glycosylated hemoglobin; total cholesterol; HDL cholesterol; and triglycerides.

### 2.3. Analytic strategy

We conducted analyses using observed variables in Mplus software (Muthen and Muthen, 1998). Statistical models were defined in the preregistration to address each primary research question. Separate models were created for our two measures of adolescent adversity: economic hardship and perceived discrimination. For each statistical model, we assessed model assumptions and made appropriate data adjustments (e.g., transformations) in cases of violations. Results did not change when skewed data were transformed to address non-normality; therefore, values are presented based on original, untransformed data. For research questions where the same model is tested but with a different adversity measure, we conducted Bonferroni family-wise error rate correction to control for multiple testing. For a power analysis, see Supplement Section S1. Additional supplementary analyses are described in Supplement Section S2.

**Fig. 1.**
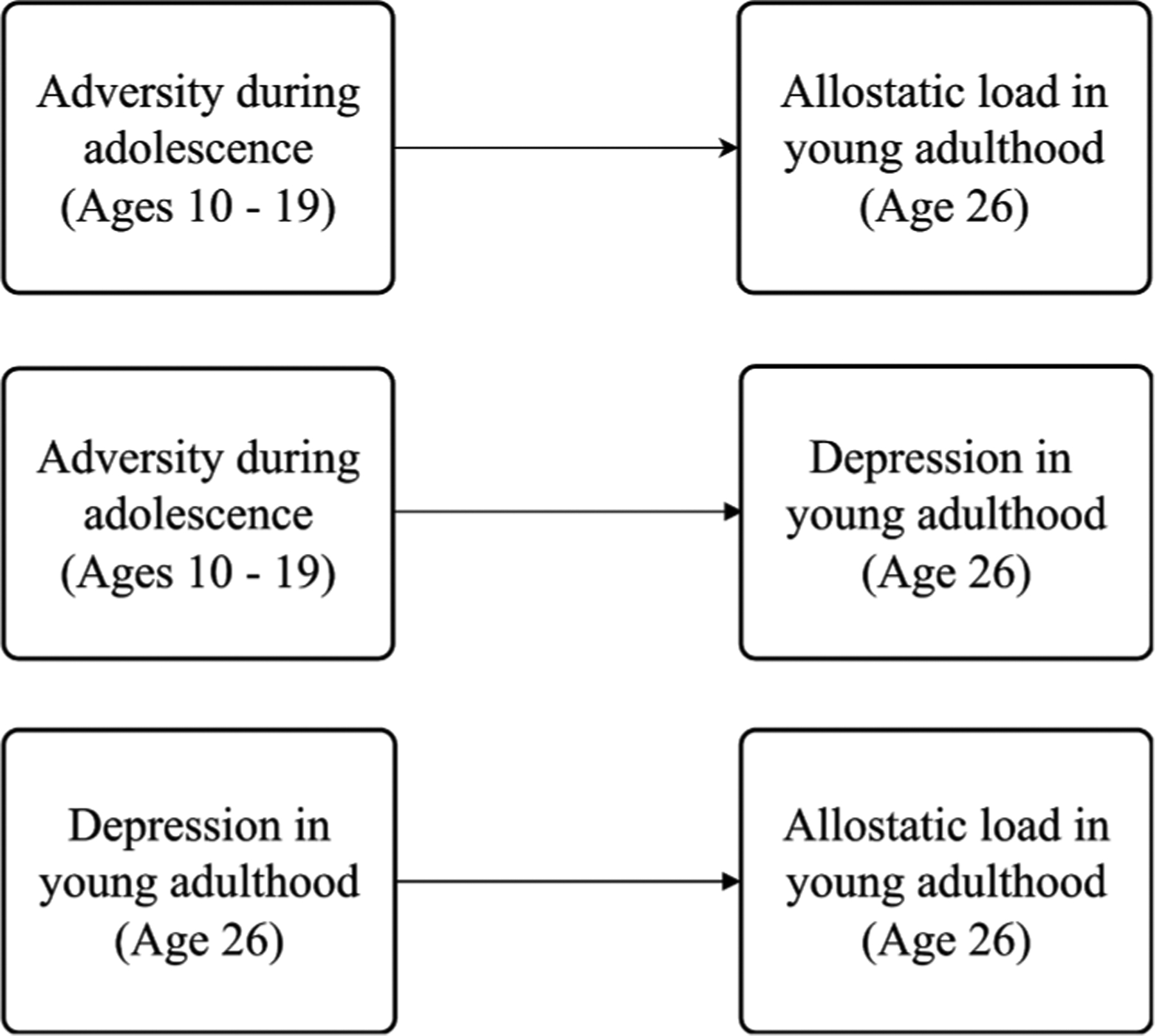
Main effects models of associations between adolescent adversity and young adult depression and allostatic load.

To test the hypothesized main effects, we constructed models (Fig. 1) in which average adversity across adolescence (ages 10 – 19) predicted AL and separately, depression symptoms at age 26. In addition, we examined the association between AL and depression at age 26 to test our hypothesis that they are positively associated. Sex was coded as binary (0 = female, 1 = male) and was controlled for in all analyses.

**Fig. 2.**
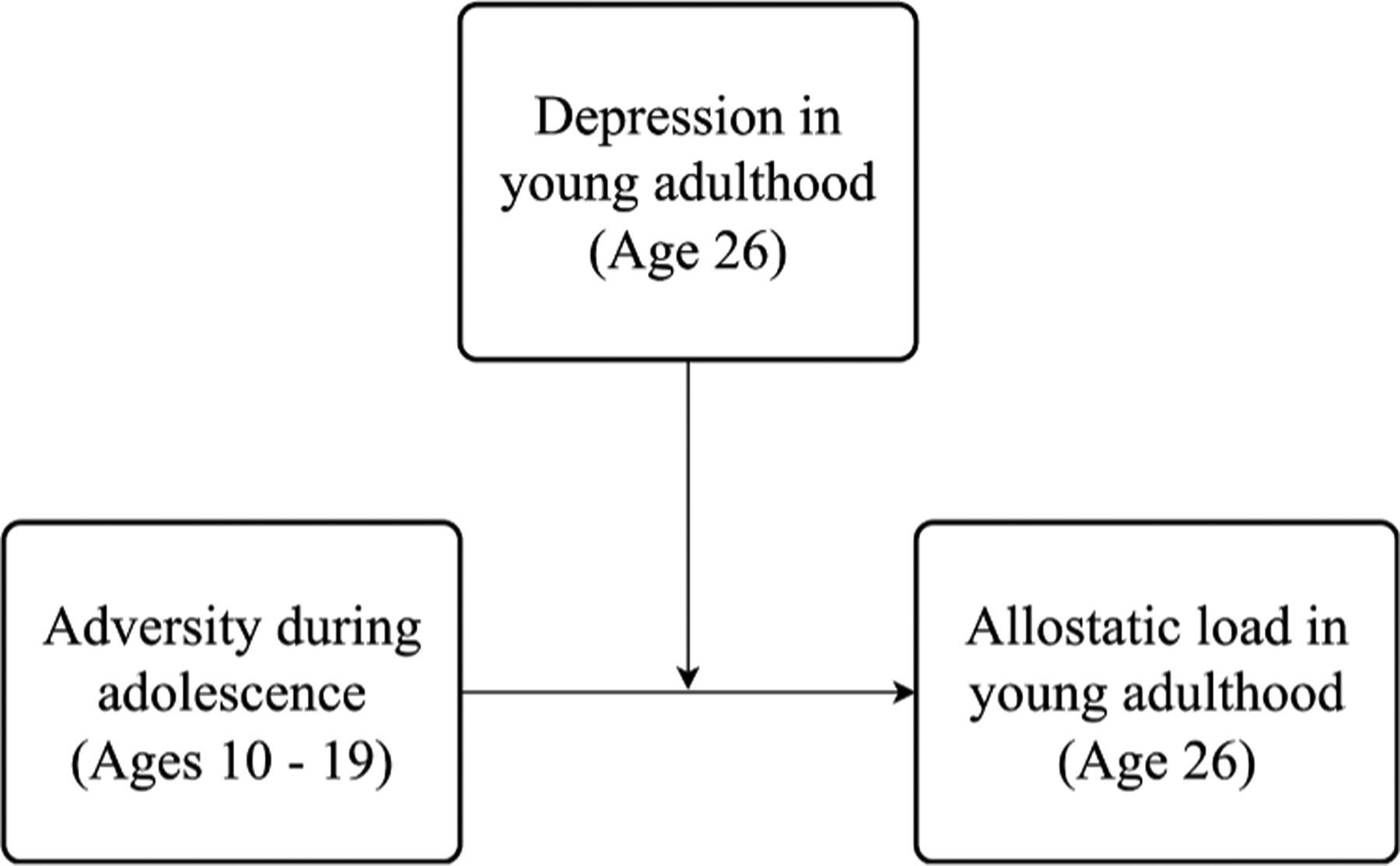
Moderation model of young adult depression moderating the association between adolescent adversity and young adult allostatic load.

To test the ‘resilience is skin deep’ hypothesis using a moderation model, we constructed a model to test whether low levels of depression at age 26 in the context of high exposure to adolescent adversity was associated with higher AL. To test this hypothesized moderation effect, we constructed models (Fig. 2) in which average adversity across adolescence (ages 10 – 19) interacted with age 26 depression symptoms to predict AL at age 26 using the same variables as for the main effects models and again controlling for sex.

**Fig. 3.**
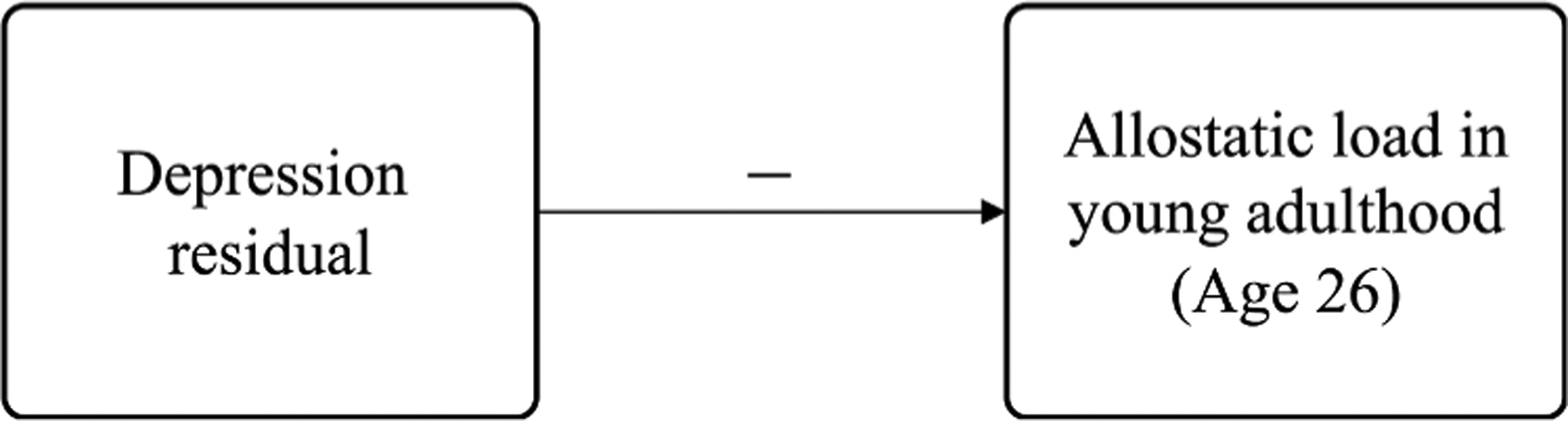
Residual scores model.

A novel, alternative method for studying resilience is to use a residual score approach. This approach has the potential to advance mechanistic resilience research by operationalizing the construct of resilience using an individual-specific and quantitative variable that is unbounded by specific definitions of resilience or specific measurement tools (Cahill et al., 2022). Therefore, we also chose to test the ‘resilience is skin deep’ hypothesis with a residual score approach to investigate whether results would differ from the statistical moderation approach when using an adversity-specific measure of resilience to predict AL. To do so, we constructed the corresponding models in two steps. First, we regressed age 26 depression symptoms onto average adversity across adolescence and saved the residual scores from this analysis to represent psychological resilience. Positive scores represented higher depression than estimated by the model (‘low resilience’), and negative scores represented lower depression than estimated by the model (‘high resilience’). Then in the second step, we constructed a regression model (Fig. 3) to test for the association between resilience to adolescent adversity (as represented by the residual scores) and age 26 AL, controlling for sex.

As in any longitudinal study, there was attrition and missing data at each wave of data collection in the CFP. To address this issue, we limited analyses to participants who participated in the age 26 assessment (see Table 1 for sample sizes available for this assessment) and excluded participants missing data on more than half the waves of the adversity measures (economic hardship and perceived discrimination), as described above. Then, we used listwise deletion for participants missing data on the summary predictor and outcome variables. Final sample sizes for each main analysis are reported in Table S4.

## 3. Results

### 3.1. Descriptive analyses

See Table 1 for descriptive statistics and Table 2 for bivariate correlations among all variables of interest. Table S3 reports descriptive statistics for the AL biomarkers.

### 3.2. Main effects model: Associations among adolescent adversity, young adult depression symptoms, and young adult AL

All model results presented herein controlled for sex. Unless specified otherwise, results described below remained the same when broadening the sample to participants with at least 4 biomarkers (Supplement Section S5). All *p*-values reported are raw, uncorrected *p-*values.

#### 3.2.1. Effects of adolescent discrimination on depression and AL at age 26

Greater perceived discrimination in adolescence significantly predicted higher levels of depression at age 26 (β =.09, *p* = .045, R^2^ =.03, ΔR^2^ = 0.005, 95% CI [.002,.181]; Figure S1). The effect of adolescent discrimination did not remain significant when correcting for multiple comparisons, when controlling for age 26 AL or age 10 depression symptoms, nor when examining associations with individual depression measures (Supplement Section S4). Additionally, perceived discrimination in adolescence did not significantly predict AL at age 26 (β = −.05, *p* = .46, R^2^ =.09, ΔR^2^ = 0.008, 95% CI [−.17,.08]). These findings partially support our hypotheses, suggesting that experiences of ethnic discrimination in adolescence elevate risk for depression, but not AL, in young adulthood. However, this discrimination-related risk for young adult depression may be partially driven by early adolescent symptoms.

#### 3.2.2. Effects of adolescent economic hardship on depression and AL at age 26

Adolescent economic hardship did not significantly predict AL (β = −.02, *p* = .74, R^2^ =.08, ΔR^2^ = 0.004, 95% CI [−.15,.11]) or depression levels (β =.04, *p* = .42, R^2^ =.03, ΔR^2^ = 0.003, 95% CI [−.05,.13]) at age 26. Contrary to our hypotheses, we did not find evidence that experiences of economic hardship in adolescence independently affect levels of depression or AL in young adulthood.

#### 3.2.3. Association between depression and AL at age 26

Depression and AL were not significantly associated at age 26 (β = −.14, *p* = .06, R^2^ =.02, ΔR^2^ = 0.002, 95% CI [−.284,.004]). Therefore, we did not find support for our hypothesis that there would be a positive association between depression and AL in young adulthood.

### 3.3. Moderation model: Young adult psychological ‘resilience’ moderating the association between adolescent adversity and young adult AL

The interaction between age 26 depression symptoms and adolescent discrimination was not significantly associated with age 26 AL (β = −.08, *p* = .21, R^2^ =.11, ΔR^2^ = −0.006, 95% CI [−.20,.04]). Thus, we did not find support for our hypothesis that participants with low levels of depression in young adulthood despite greater experiences of ethnic discrimination in adolescence would exhibit higher AL in young adulthood.

**Fig. 4.**
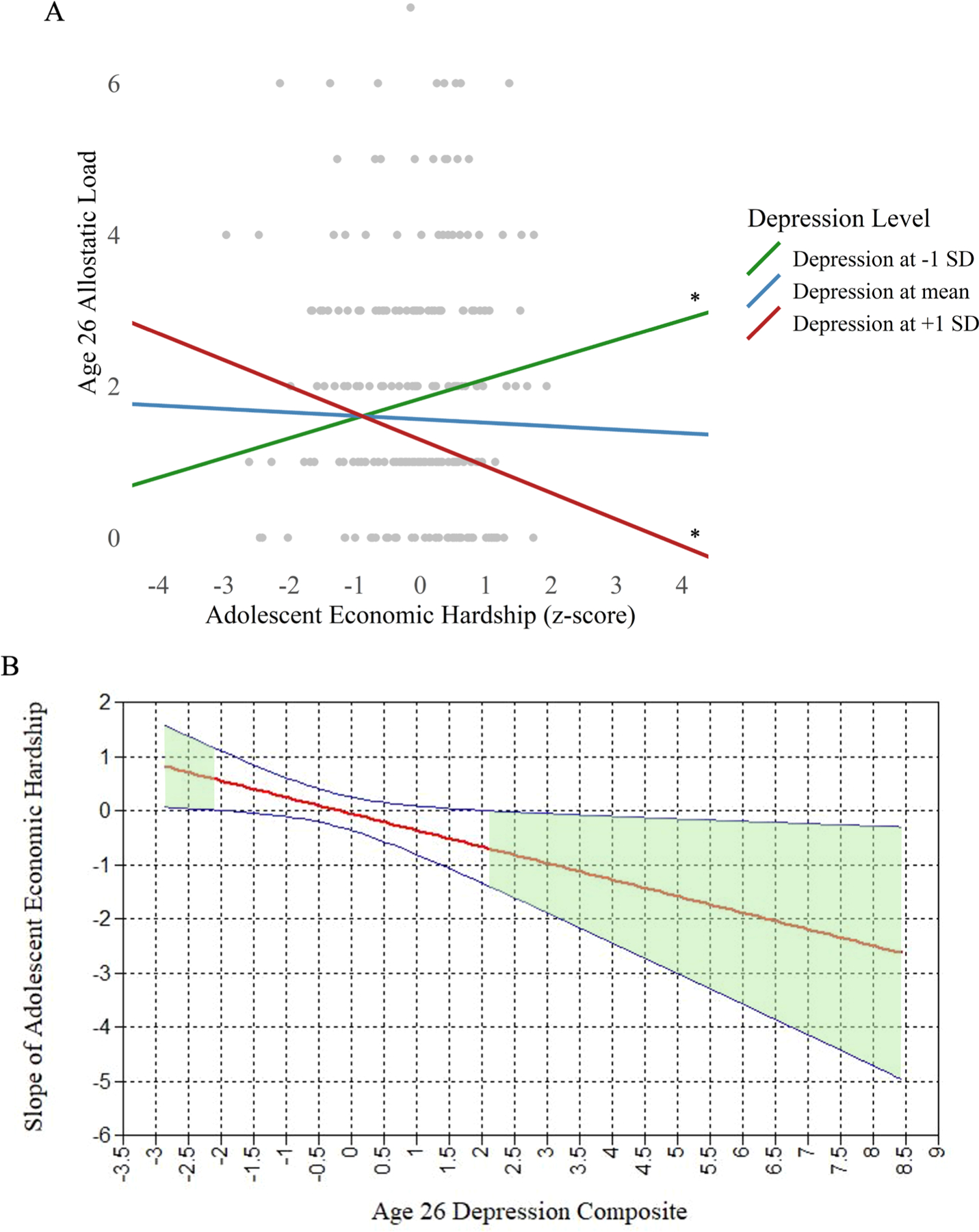
Interaction between adolescent economic hardship and age 26 depression in predicting AL. *Note*. A. * *p* < .05 (simple slope is significantly different from zero). B. Johnson-Neyman region of significance plot illustrating values of the age 26 depression composite (X axis) for which the adjusted slope of adolescent economic hardship in predicting age 26 AL (Y axis) is statistically significant. Regions of significance highlighted in green.

In contrast, the interaction between age 26 depression symptoms and adolescent economic hardship was significantly associated with age 26 AL (β = −.17, *p* = .03, R^2^ =.12, ΔR^2^ =.028, 95% CI [−.33, − .02]); however, this association would not survive correction for multiple comparisons and was not significant in the subsample of participants with at least 4 AL biomarkers (Supplement S5). In this model, depression at age 26 was also inversely associated with AL (β = −.16, *p* = .01, 95% CI [−.28, −.03]). Simple slopes analysis suggested that the direction of association between economic hardship and age 26 AL changed depending on age 26 depression symptoms (Fig. 4A). Greater adolescent economic hardship was significantly positively associated with higher AL at lower levels of depression (1 SD below the sample mean; *b* =.68, *p* = .04, 95% CI [.03, 1.32]). In contrast, we observed a nonsignificant, but negative, association between economic hardship at average levels of depression (at the sample mean; *b* = −.05, *p* = .76, 95% CI [−0.35, 0.26]), and a significant, negative association between economic hardship and AL at higher levels of depression (at 1 SD above the sample mean; *b* = −.78, *p* = .04, 95% CI [−1.53, −0.02]). The Johnson-Neyman procedure revealed that economic hardship significantly predicted AL at age 26 for depression composite values at or below −2.1 (0.88 SD below mean), and at or above 2.1 (0.95 SD above mean) (Fig. 4B). These findings support the ‘resilience is skin deep’ hypothesis for economic adversity. Specifically, participants with low levels of depression in young adulthood despite high levels of economic hardship in adolescence exhibited higher AL in young adulthood. However, this result should be interpreted with caution given that this analysis was underpowered and results would not survive correction for multiple comparisons.

### 3.4. Residual scores model: Associations between ‘resilience’ to adolescent adversity and young adult AL

Psychological resilience to adolescent discrimination (i.e., lower depression symptoms at age 26 despite higher discrimination in adolescence) was not significantly associated with age 26 AL (β = −.13, *p* = .06, R^2^ =.10, ΔR^2^ =.022, 95% CI [−.269,.006]). However, when broadening the sample to participants with at least 4 biomarkers, psychological resilience to adolescent discrimination was significantly associated with higher age 26 AL (see Supplement S5). Resilience to adolescent economic hardship (i.e., lower depression symptoms at age 26 despite higher economic hardship in adolescence) was not significantly associated with age 26 AL (β = −.12, *p* = .09, R^2^ =.09, ΔR^2^ =.017, 95% CI [−.25,.02]). In sum, when defining resilience using a residual scores approach, we only found support for the ‘resilience is skin deep’ hypothesis for experiences of ethnic discrimination when including participants with at least 4 biomarkers.

### 3.5. Moderation by sex

Sex did not significantly moderate any of the pathways in the main effects, moderation, or residual scores models (See Supplement Section S3). Because no significant moderation effect was found, results suggest that pathways between adversity, depression, and AL did not differ for men and women.

## 4. Discussion

Intersectionality frameworks emphasize the health risk disparities evident for individuals who experience multiple sources of social stratification. The goal of the present study was to test two models of the association between discrimination and economic hardship in adolescence and depression symptoms and AL in young adulthood in a sample of Mexican-origin women and men. Overall, we found partial support for the ‘resilience is skin deep’ hypothesis, adding to our understanding of how developmental adversity affects later health and well-being in this population.

Consistent with the ‘skin-deep resilience’ hypothesis, we observed a tradeoff in health outcomes for Latino young adults with developmental histories of high economic adversity. Specifically, high levels of economic hardship predicted higher AL in young adulthood for those with low levels of depression. This finding suggests that psychological resilience to chronic poverty across the teenage years may come at the cost of additional physical strain resulting from high-effort adaptation to cumulative stress. While this preliminary interaction effect would not survive correction for multiple comparisons, similar tradeoffs have been found in previous longitudinal studies of skin-deep resilience in which high-effort coping to socioeconomic disadvantage (e.g., better self-control, college completion) predicted lower levels of depressive symptoms but also greater signs of stress-related physiological strain, including AL (Brody et al., 2020, 2013; Chen et al., 2015; Gaydosh et al., 2018; Miller et al., 2020, 2015). Importantly, we tested this effect in a sample of Latino individuals, who to our knowledge have been underrepresented in skin-deep resilience research.

Surprisingly, we found evidence for a more straightforward link between mental and physical health for young adults with developmental histories of economic security. Specifically, lower economic hardship in adolescence also predicted higher AL scores for young adults with greater symptoms of depression. It is worth noting that these individuals grew up in working class families that were largely lower middle class. While severe family financial strain was not their primary experience of developmental adversity, it is possible that they were exposed to other adversities not captured in this study that elevated their risk for both depression and high AL. Further research is needed to identify other sources of adversity in this subpopulation and replicate the preliminary result of this exploratory analysis, given that the moderation effect did not survive correction for multiple comparisons.

Our hypotheses for the effects of ethnic discrimination in adolescence on health and well-being in young adulthood received partial support. As predicted, we found that greater perceived discrimination across adolescence predicted higher levels of depression at age 26, which aligns with prior work. Meta-analyses have shown robustly detrimental effects of racial discrimination on depression and other mental health problems in adolescence (Benner et al., 2018). Previous research with the CFP dataset similarly found that peer discrimination early in adolescence predicted increased symptoms of depression and anxiety in participants’ senior year of high school (Stein et al., 2019). The current study demonstrated that these effects may extend into emerging adulthood, a time of uncertainty when many young people explore more adult-like identities and responsibilities before committing to stable life trajectories (Arnett et al., 2014). However, this preliminary finding would not survive correction for multiple comparisons; therefore, it should be interpreted with caution.

Discrimination in adolescence did not, however, predict higher AL in young adulthood, contrary to our hypothesis. It is possible that physical health effects emerge later in life after discriminatory experiences accumulate over time, or when physical health problems become more pronounced and prevalent. In contrast, coping with the strain of economic hardship may have had an impact on physical health for these young adults due to financial barriers to accessing quality healthcare and nutritious food. Supporting this idea, results from the Add Health study have shown that financial hardship during childhood and adolescence has a cumulative impact on physical health in early adulthood (Wickrama et al., 2015).

Moreover, we did not find sex differences in associations among adversity, mental well-being, and physical health in this study. When examining ethnic discrimination, the association between adolescent experiences of discrimination and young adult depression did not differ by sex. However, we did observe a small, significant association between sex and discrimination (Table 2) indicating that males reported higher levels of ethnic discrimination than females. This pattern maps onto findings in the literature suggesting that Latino men face greater experiences of discrimination than women, though whether the corresponding risk of mental health problems differs by gender remains unclear (Lazarevic et al., 2018). The lack of sex differences in the link between ethnic discrimination and internalizing symptoms such as depression in this sample may stem from the tendency for boys to be at a higher risk of externalizing problems whereas girls are at a higher risk of internalizing problems (Nair et al., 2013). Indeed, we observed a significant link between sex and depression symptoms in young adulthood indicating higher symptoms of depression in females.

### 4.1. Limitations and future directions

Strengths of this study were that we examined how distinct exposures to adversity impacted mental and physical health over time in a sample of Mexican-origin young adults. However, our study was not without limitations. First, we did not test whether discrimination and economic hardship across adolescence interact to predict depression and AL in young adulthood. As such, this is an important direction for future research. According to an intersectionality framework, we expect the combined effect of higher levels of both discrimination and economic hardship to have the strongest negative impact on health.

Future work should also expand on this study’s methodology of mental health outcomes. We used a composite measure of depression that relied on self-report, which introduces potential biases stemming from social desirability, memory limitations, or cultural differences. Accordingly, these results should be replicated using clinician ratings of depression. Additionally, while we focused on depression as a form of stress-related psychopathology in this study, it is possible that other mental health issues such as anxiety, post-traumatic stress disorder, or externalizing disorders may be more salient for this group.

Additionally, we did not examine other sources of complexity in the link between health and marginalization based on social characteristics. For example, participants in our sample who were immigrants and/or sexual minorities may have experienced added developmental adversities beyond experiences of ethnic discrimination and economic hardship. Indeed, in this issue, Vargas et al. (2025) found greater hair cortisol concentrations in individuals who reported intersectional racism and heterosexism as opposed to either type of discrimination alone. Moreover, while we used moderation and residual scores approaches to capture psychological resilience to adolescent adversity, we did not account for resilience-promoting factors in our models, which may have helped explain null effects. Indeed, prior work in the CFP sample has found that higher levels of ethnic pride in late adolescence predicted lower levels of depression symptoms at ages 21 and 23 (Carranza et al., 2025). As such, future research should measure additional sources of marginalization, privilege, and resilience to capture more nuance in longitudinal associations between adversity and health.

An important qualification to our AL results is that, out of the full sample, fewer participants agreed to blood collection than originally anticipated. This introduces methodological limitations to our AL index, which primarily relied on blood biomarkers. First, the resulting sample is likely biased, as participants that were especially healthy or saw doctors more frequently may have been more likely to agree to the blood test. Second, our AL analyses were underpowered, especially to detect moderation effects, and the significant moderation effect we observed did not survive correction for multiple comparisons. Therefore, main effects of adolescent ethnic discrimination and economic hardship on young adult AL as well as the skin-deep resilience effects tested in the present study should be examined further in a larger sample.

In addition, our AL index did not include a neuroendocrine biomarker, such as cortisol, epinephrine, or dehydroepiandrosterone. Neuroendocrine function is a key component of the stress response and thus central to the stress-related multisystemic dysregulation explained by the AL model (McEwen, 1998). Therefore, these results will need to be replicated in other samples using an AL index that includes a measure of neuroendocrine function along with biomarkers for the other systems implicated in the AL model (e.g., immune, metabolic, and cardiovascular) that were included in this study. However, our hypotheses for the AL index we pre-registered were informed by a previous study of socioeconomic adversity and AL in Latino youth using a similar index that also did not include a neuroendocrine biomarker (Gallo et al., 2019).

Finally, one way in which we tested the ‘resilience is skin deep’ hypothesis was using a moderation analysis where the interaction between adolescent adversity and age 26 depression predicted AL at the same time point at which depression was assessed, limiting causal inference. Because the depression-AL associations were cross-sectional, directionality of this link is uncertain. Therefore, it is unclear whether high-effort coping and low depression across adolescence was the cause of higher AL in young adulthood in our skin-deep resilience model. Future studies should examine this question using assessments of adversity, depression, and AL at multiple timepoints to clarify the direction of influence.

### 4.2. Conclusions

In summary, this study contributes to our understanding of the effects of two forms of developmental adversity, discrimination and economic hardship, on health and well-being in Mexican-origin emerging adults. In particular, results of the moderation model suggest that the strain associated with coping with chronic adversity such as economic hardship may come at a cost to physical health, although these results should be considered preliminary until confirmed with larger samples. Therefore, intervention work that benefits Latino adolescents and promotes sustainable, holistic resilience may help mitigate the detrimental impacts of ethnic discrimination and economic hardship on their adult development.

## Supplementary Material

1

2

3

4

5

## Figures and Tables

**Table 1:** Descriptive statistics.

Variable	*n*	*M*	*SD*	Min	Max
*Age 10–19 Variables*					
Discrimination	610	1.19	0.15	1.00	2.17
Economic Security	608	6.80	1.22	3.77	9.87
Income-to-Needs	606	1.32	0.81	0.15	6.13
*Age 26 Variables*					
DIS-5 Major Depression Symptoms	503	2.11	3.28	0	9
MASQ General Distress	507	1.47	0.64	1	4
MASQ Anhedonic Depression	507	2.06	0.71	1	4
Composite Depression Score	503	0.006	2.38	−2.87	8.45
Allostatic Load	217	1.97	1.66	0	7

*Note*: This table reports unstandardized values. DIS-5: Diagnostic Interview Schedule for DSM-5. MASQ: Mood and Anxiety Symptom Questionnaire. The composite depression score was calculated by first standardizing the average MASQ General Distress and Anhedonic Depression subscale scores and standardizing the DIS-5 Major Depression module symptom counts and then summing these standardized measures.

**Table 2:** Bivariate correlations among study variables.

Variable	1	2	3	4
1. Sex (0 = female, 1 = male)	—			
2. Adolescent Discrimination	.08*	—		
3. Adolescent Economic Hardship	.02	.05	—	
4. Age 26 Depression	−.16**	.07	.03	—
5. Age 26 Allostatic Load	.28**	−.02	−.03	−.15*

*Note*: Adolescent variables spanned ages 10–19 years.

* *p* < .05.

** *p* < .01.

## Data Availability

The raw data from this study cannot be shared because participants did not consent to the public sharing of data. However, the data that supports the findings of this study are available on request from the corresponding author.

## Footnotes

1 To remain consistent with community preference and the Spanish language, we will use “Latino” as a gender-neutral descriptor for Hispanic people of Latin American origin (Hussain et al., 2021; Noe-Bustamante et al., 2020).

2 We use the term “Mexican origin” to refer to individuals or families who identify as being of Mexican heritage.

## Appendix A. Supporting information

Supplementary data associated with this article can be found in the online version at doi:10.1016/j.psyneuen.2026.107832.
