# Supplementary material for: Associations between adolescent adversity and young adult depression symptoms and allostatic load in Mexican-origin individuals
Source: Psychoneuroendocrinology. Author manuscript; Available in PMC 2026 Jun 24. (PMC13293614; doi:10.1016/j.psyneuen.2026.107832)
Supplement: 1 [file NIHMS2180036-supplement-1.docx]

**Supplementary Material**

**Appendix S1.** Power Analysis.

A priori power analyses were conducted in G*Power version 3.1.9.6 (Faul et al., 2007) to determine adequate sample sizes for testing the models described in the main text. To account for publication bias, we based power analyses on the lowest available or lowest meaningful estimates of effect sizes. Effect size interpretations were based on Cohen’s (1988) effect size guidelines.

*Main effects*

The main effects models tested whether adolescent adversity, specifically economic hardship and perceived discrimination, predicts young adult depression and allostatic load (AL), controlling for sex. Previous research on associations between economic hardship and AL (Evans and De France, 2022) suggests a small effect (*r =* .17). Similarly, a meta-analysis reporting associations between economic hardship and depression (Lorant et al., 2003) suggests small effect sizes (*r =* .14). While previous research on perceived discrimination and AL is somewhat limited, existing work (Upchurch et al., 2015) suggests small effect sizes (*r =* .17). Similarly, prior work on discrimination and depression symptoms in Mexican-origin adults (Flores et al., 2008) suggests small effect sizes (*r* = .26). Finally, prior research on associations between allostatic load and depression in adults (Gale et al., 2015) again suggests small effect sizes (*r* = 0.17).

The above main effect models, each including two predictors and one outcome, were tested using multiple regression. Conservative estimates of f^2^ effect sizes for multiple regression models were calculated in G*Power for each main effect model based on previously reported bivariate correlation coefficients in the literature, as described above. These estimated f^2^ effect sizes ranged from 0.02 to 0.06, representing small effects (Cohen, 1988). Using a significance criterion of *α* = .05 and 80% power, the minimum sample size needed to detect a small f^2^ effect of 0.02 in a linear multiple regression model with 2 predictors testing for *R*^2^ deviation from zero is 485.

*Moderation effects*

The moderation model tested whether adolescent adversity, specifically economic hardship and perceived discrimination, interacted with young adult depression to predict young adult AL, controlling for sex. These models were also tested with multiple regression, and in each model, there were 4 predictors of AL: the main effect of depression, the main effect of adversity, the interaction effect of depression x adversity, and the controlled effect of sex. Prior literature testing similar moderation models is sparse; however, one study by Brody et al. (2013) reported a significant moderation effect for a model testing whether cumulative socioeconomic status risk at ages 11 - 13 interacted with self-control to predict AL at age 19. Taking the *R*^2^ for the full model including the interaction (.043) and subtracting it from the previous model without the interaction (.028) results in a partial *R*^2^ of .015 for the interaction. This translates to a small f^2^ effect size of 0.02 using the conversion formula $f^{2}= \frac{R^{2}}{(1- R^{2})}$. Using a significance criterion of *α* = .05 and 80% power, the minimum sample size needed to detect this small f^2^ effect in a linear multiple regression model with 4 predictors testing for an *R*^2^ increase is 485.

*Resilience effects*

The third model tested whether psychological resilience to adolescent adversity, as reflected by lower residual scores in a regression model with adolescent adversity predicting young adult depression, would be associated with young adult AL, controlling for sex. Using a similar method in middle-aged adults, Linneman et al. (2022) found a medium effect size for the association between residual scores (i.e., psychological resilience to stressors) and self-rated health (*r* = -0.49). Another study in older adults examining the association between resilience as measured by the Brief Resilience Scale and health as indexed by chronic disease comorbidities found a correlation of *r* = -.32 (da Silva-Sauer et al., 2021). The residual scores models for perceived discrimination and economic hardship, each including two predictors and one outcome, were tested using multiple regression. Assuming smaller effect sizes with our young adult sample (*r* ~= .16), we calculated a conservative estimate of small f^2^ effect size for a multiple regression model in G*Power. Using a significance criterion of α = .05 and 80% power, the minimum sample size needed to detect a small f^2^ effect of 0.03 in a linear multiple regression model with 2 predictors testing for R^2^ deviation from zero is 325.

*Achieved power*

The sample size estimates described above were used to guide data collection efforts and the development of *a priori* hypotheses and statistical models. When beginning data collection for the age 26 assessment, we assumed based on retention in previous longitudinal waves that out of the original 674 participants in the California Families Project, we would retain approximately 80% of the sample (~540 participants). Thus, based on prior retention within the sample, we expected sufficient power to test all study aims. However, our achieved power was lower than our 80% estimate due to more participants declining to provide a blood sample than expected, which reduced the sample size available for the AL indicators. See the main text for further discussion of the limitations associated with sample size and power for the analyses.

**Appendix S2.** Supplementary Analysis Methods.

*Moderation by sex.* We examined whether sex moderates the pathways in the three types of models presented in the main text, as the prevalence of MDD is consistently found to be higher in women (e.g., Weissman et al., 1993) and sex differences have also been found in AL (e.g., Richardson et al., 2021) as well as for the association between depression and AL (e.g., Bey et al., 2018).

*Supplementary analyses for the main effects model.* For both economic hardship and perceived discrimination in adolescence, we tested whether these adversities predicted age 26 depression while controlling for age 26 AL. Similarly, we tested whether these adversities predicted age 26 AL while controlling for age 26 depression. Furthermore, we tested whether these adversities predicted an increase in depression from age 10 to 26 using the same strategy as for the main analyses but controlling for age 10 depression, as measured by the Major Depressive Disorder module of the Diagnostic Interview Schedule for Children Version IV (DISC-IV; Shaffer et al., 2000). Finally, we tested whether results from the main effects models changed when analyzing AL with a continuous approach rather than a quartile approach by standardizing and averaging all AL indices to create a continuous version of this variable.

*Supplementary analyses for the moderation model.* We tested whether the moderation effect of age 26 depression on the association between adolescent economic hardship and age 26 AL was due to concurrent economic hardship experienced at age 26. To test this research question, we controlled for concurrent (i.e., age 26) economic hardship in the moderation model using measures of income-to needs ratio and financial hardship as described in the main text, but asked of participants themselves rather than their mothers (Conger et al., 2002, 1991; Weissman et al., 2018). Similarly, we tested whether the moderation effect of age 26 depression on the association between adolescent perceived discrimination and age 26 AL was due to concurrent discrimination experienced at age 26. To test this research question, we controlled for concurrent perceived discrimination in the moderation model using sum scores from the Personal Experiences with Prejudice and Discrimination subscale of the Perceptions of Discrimination Scale as described in the main text (Johnston and Delgado, 2004). Finally, as with the main effects models, we tested whether results from the moderation model changed when analyzing AL with a continuous approach rather than a quartile approach using the same strategy described above for the supplementary main effects models.

**Appendix S4.** Supplementary Results.

*Moderation by sex.* In the main effects models, the interaction between adolescent economic hardship and sex was not significant in predicting AL (β = .02, *p* = .86, R^2^ = .08, ΔR^2^ = 0, 95% CI [-.17, .21]) or depression (β = -.04, *p* = .54, R^2^ = .03, ΔR^2^ = 0.001, 95% CI [-.17, .09]) at age 26. Similarly, the interaction between adolescent discrimination and sex was not significant in predicting AL (β = .07, *p* = .53, R^2^ = .09, ΔR^2^ = 0.001, 95% CI [.16, .41]) or depression (β = -.05, *p* = .41, R^2^ = .03, ΔR^2^ = 0.002, 95% CI [-.18, .07]) at age 26. The interaction between age 26 AL and sex was also not significant in predicting age 26 depression (β = -.06, *p* = .56, R^2^ = .02, ΔR^2^ = 0.001, 95% CI [-.26, .14]).

In the moderation models, the 3-way interaction between sex, adolescent discrimination, and depression was not significant in predicting age 26 AL (β = -.22, *p* = .09, R^2^ = .12, ΔR^2^ = 0.013, 95% CI [-.47, .04]). The 3-way interaction between sex, adolescent economic hardship, and depression also did not significantly predict age 26 AL (β = -.10, *p* = .34, ΔR^2^ = 0.005, R^2^ = .13, 95% CI [-.31, .10]).

In the residual score models, the interaction between resilience to adolescent economic hardship (residual scores of age 26 depression) and sex was not significant in predicting age 26 AL (β = -.09, *p* = .36, R^2^ = .10, ΔR^2^ = 0.004, 95% CI [-.28, .10]). Similarly, the interaction between resilience to adolescent discrimination (residual scores of age 26 depression) and sex was not significant in predicting age 26 AL (β = -.07, *p* = .48, R^2^ = .10, ΔR^2^ = 0.002, 95% CI [-.26, .12]).

*Supplementary analyses for the main effects model.* Adolescent perceived discrimination did not predict age 26 depression above and beyond age 26 AL: controlling for both sex and age 26 AL, adolescent discrimination no longer significantly predicted depression at age 26 (β = .12, *p* = .12, ΔR^2^ = 0.012, R^2^ = .03, 95% CI [-.03, .26]). Similarly, adolescent discrimination did not significantly predict age 26 AL above and beyond age 26 depression (β = -.03, *p* = .63, ΔR^2^ = 0.007, R^2^ = .10, 95% CI [-.15, .09]). Adolescent economic hardship also did not significantly predict AL at age 26 above and beyond depression at age 26 (β = .05, *p* = .47, R^2^ = .02, ΔR^2^ = 0, 95% CI [-.09, .20]). Moreover, adolescent economic hardship did not significantly predict depression at age 26 above and beyond age 26 AL (β = -.01, *p* = .84, R^2^ = .09, ΔR^2^ = 0.001, 95% CI [-.15, .12]).

For tests of whether adolescent adversity predicted increases in depression from age 10 to age 26, we controlled for age 10 depression in models predicting age 26 depression. Compared to the results presented in the main text, adolescent perceived discrimination no longer significantly predicted age 26 depression (β = .06, *p* = .19, R^2^ = .04, ΔR^2^ = 0.005, 95% CI [-.03, .16]). In this model, the small positive association between age 10 depression and age 26 depression was significant (β = .13, *p* = .005, 95% CI [.04, .22]), suggesting that symptoms of depression early in adolescence may have been driving the association between adolescent discrimination and young adulthood depression symptoms. Similarly, controlling for both sex and age 10 depression, adolescent economic hardship did not significantly predict age 26 depression (β = .01, *p* = .76, R^2^ = .04, ΔR^2^ = 0.003, 95% CI [-.08, .11]). In this model, the small positive association between age 10 depression and age 26 depression was also significant (β = .12, *p* = .007, 95% CI [.03, .21]).

Lastly, results from the main effects model did not change when analyzing AL with a continuous approach rather than a quartile approach. Adolescent economic hardship did not significantly predict continuous AL at age 26 (β = .05, *p* = .45, R^2^ = .10, ΔR^2^ = 0.006, 95% CI [-.08, .17]), nor did adolescent perceived discrimination (β = -.02, *p* = .78, R^2^ = .09, ΔR^2^ = 0.003, 95% CI [-.15, .11]). However, depression and continuous AL were inversely associated at age 26 (β = -.17, *p* = .007, R^2^ = .03, ΔR^2^ = 0.025, 95% CI [-.29, -.05]), suggesting a general tradeoff between mental and physical well-being in young adulthood when using a continuous measure of AL.

*Supplementary analyses for the moderation model.* Results of the moderation models did not change when accounting for concurrent adversity. Controlling for sex and concurrent economic hardship at age 26, the interaction between adolescent economic hardship and age 26 depression was still significantly associated with age 26 AL (β = -.17, *p* = .04, R^2^ = .12, ΔR^2^ = 0.03, 95% CI [-.57, -.02]). In this model, the small positive association between concurrent economic hardship and AL was not significant (β = .05, *p* = .52, 95% CI [-.10, .20]).

For perceived discrimination, controlling for sex and concurrent discrimination at age 26, the interaction between adolescent discrimination and age 26 depression was still not significantly associated with age 26 AL (β = -.07, *p* = .23, R^2^ = .11, ΔR^2^ = 0.004, 95% CI [-.19, .05]). In this model, the small negative association between concurrent discrimination and AL was also non-significant (β = -.06, *p* = .34, 95% CI [-.17, -.06]).

Lastly, results of the moderation models partially changed when analyzing AL with a continuous approach rather than a quartile approach. The interaction between age 26 depression symptoms and adolescent economic hardship was not significantly associated with continuous age 26 AL (β = -.05, *p* = .51, R^2^ = .12, ΔR^2^ = 0.002, 95% CI [-.18, .09]), in contrast to the significant interaction described in the main text. However, the interaction between age 26 depression symptoms and adolescent discrimination was still not significantly associated with continuous age 26 AL (β = -.05, *p* = .42, R^2^ = .12, ΔR^2^ = 0.002, 95% CI [-.18, .08]).

**Appendix S5.** Results of Sensitivity Analyses.

*Allostatic Load.* In the main effects models, for participants with at least 4 biomarkers, adolescent economic hardship was not significantly associated with age 26 AL (β = -.02, *p* = .77 R^2^ = .06, ΔR^2^ = 0.002, 95% CI [-.14, .10]). Similarly, perceived discrimination in adolescence did not significantly predict age 26 AL (β = -.06, *p* = .24 R^2^ = .06, ΔR^2^ = 0.01, 95% CI [-.17, .04]). As in the primary results, depression was not significantly associated with AL at age 26 for this subsample (β = -.07, *p* = .27 R^2^ = .02, ΔR^2^ = 0.008, 95% CI [-.19, .05]).

In the moderation models, the interaction between age 26 depression symptoms and adolescent discrimination was still not significantly associated with age 26 AL (β = -.08, *p* = .17, R^2^ = .07, ΔR^2^ = .005, 95% CI [-.19, .03]) for participants with at least 4 biomarkers. However, in contrast to the primary analysis results described in the main text, the interaction between age 26 depression symptoms and adolescent economic hardship for participants with at least 4 biomarkers was not significant (β = -.12, *p* = .08, R^2^ = .07, ΔR^2^ = .013, 95% CI [-.24, .01]).

In the residual score models, psychological resilience to adolescent economic hardship (i.e., lower depression symptoms at age 26 despite higher economic hardship in adolescence) was not significantly associated with age 26 AL (β = -.06, *p* = .36 R^2^ = .06, ΔR^2^ = 0.002, 95% CI [-.17, .06]) for participants with at least 4 biomarkers. However, for this broadened sample, psychological resilience to adolescent discrimination (i.e., lower depression symptoms at age 26 despite higher discrimination in adolescence) was significantly associated with higher age 26 AL (β = -.17, *p* = .03 R^2^ = .12, ΔR^2^ = 0.067, 95% CI [-.30, .02]).

*Depression.* As described in the main text, greater perceived discrimination in adolescence significantly predicted increased levels of depression at age 26 using the composite score. Therefore, we tested whether results changed when modeling adolescent perceived discrimination as a predictor of each of the three depression measures separately. Results changed when analyzing individual measures: Adolescent discrimination did not significantly predict DIS-5 MDD module symptom counts (β = .05, *p* = .30, R^2^ = .02, ΔR^2^ = 0.001, 95% CI [-.05, .16]) or MASQ Anhedonic Depression scores (β = .05, *p* = .20, R^2^ = .02, ΔR^2^ = 0.001, 95% CI [-.13, .61]), but still significantly predicted MASQ General Distress scores (β = .11, *p* = .02, R^2^ = .03, ΔR^2^ = 0.009, 95% CI [.02, .21]) at age 26. These results suggest that the association between adolescent discrimination and adult depression was driven by general distress scores.

**Supplementary Tables**

| Table S1 | | | | | | | |
| --- | --- | --- | --- | --- | --- | --- | --- |
| *Preregistration Deviations* | | | | | | | |
| **Deviations** | | | | | | | |
| # | Details | | Original Wording or Plan | Deviation Description | | Reader Impact | |
| 1 | Type | Variables | We planned to include total hemoglobin levels obtained from blood samples as one of the 9 biomarkers in our AL measure. | We did not obtain total hemoglobin. Total hemoglobin tests for anemia; however, we did not have hypotheses about anemia, and this biomarker would require a second blood draw. Therefore, we had decided not to measure it. This biomarker was included in the preregistration in error and the number of biomarkers in the AL measure was 8 rather than 9. | | This deviation has a minor impact on readers’ interpretations of our results. It resulted from an oversight when writing the preregistration and was corrected prior to data analysis, when preparing the data. Because we did not already know the results when we made this correction, the risk of bias is low. The small difference in interpretation of results is that our AL measure does not reflect anemia. | |
|  | Reason | Miscommunication |  |  |  |  |  |
|  | Timing | After data access |  |  |  |  |  |
| 2 | Type | Variables | The Financial Cutbacks scale consisted of **7 items** asking about their adjustments to financial need (1 = yes, 0 = no) during the past 3 months. | This scale has 9 items. The number of items described in the preregistration was an error. | | This deviation has minimal impact on readers’ interpretations of the results. It may lead to misunderstandings of the extent to which our economic hardship composite score reflected financial cutbacks. The risk of bias is low because we recognized this error before analyzing the data. | |
|  | Reason | Typo/Error |  |  |  |  |  |
|  | Timing | After data access |  |  |  |  |  |
| 3 | Type | Data Preparation | The Financial Cutbacks scale consisted of 7 items asking about their adjustments to financial need (**1 = yes, 0 = no**) during the past 3 months. | This scale was coded in the dataset as 1 = yes, 2 = no. | | This deviation has minimal impact on readers’ interpretations of our findings. We miscommunicated the way this variable was coded in data entry in the preregistration. | |
|  | Reason | Typo/Error |  |  |  |  |  |
|  | Timing | After data access |  |  |  |  |  |
| 4 | Type | Data Preparation | To quantify depression symptoms, we will create a single composite variable by standardizing the measures of symptom counts from the major depression module of the DIS-5 as well as the Anhedonic Depression and General Distress subscales of the MASQ and then summing the standardized measures. | We averaged the MASQ subscales rather than computing symptom counts to be consistent with prior publications on this dataset. | | This deviation has minimal impact on readers’ interpretations of our findings. The MASQ subscale scores should have been described as averages in the preregistration. | |
|  | Reason | Typo/Error |  |  |  |  |  |
|  | Timing | After results known |  |  |  |  |  |
| 5 | Type | Data Preparation | For analyses, we will use sum scores from the Personal  Experiences with Prejudice and Discrimination subscale that asks about adolescents’ personal experiences with discrimination. | We averaged scores from the Personal Experiences with Prejudice and Discrimination subscale within each wave to be consistent with prior publications on this dataset. | | This deviation has minimal impact on readers’ interpretations of our findings. These subscale scores should have been described as averages in the preregistration. | |
|  | Reason | Typo/Error |  |  |  |  |  |
|  | Timing | After results known |  |  |  |  |  |
| 6 | Type | Other (Please Explain) | For research questions where the same model is tested but with a different adversity measure, we wrote that we would conduct a false discovery rate correction to control for multiple testing. | We intended to write that we would conduct the more conservative correction for family-wise error rate using the Bonferroni procedure. A false discovery rate correction would be too liberal to use with our analyses because we only conducted two statistical tests for each model, corresponding to the two forms of adversity. | | This deviation has moderate impact on readers’ interpretations of our findings, as they may expect our correction procedure to be less conservative. We specified the accurate correction procedure in the method section and clarified in the discussion that our results did not survive correction for multiple comparisons. | |
|  | Reason | Typo/Error |  |  |  |  |  |
|  | Timing | After results known |  |  |  |  |  |
| 7 | Type | Analysis | The original manuscript included participants with data on at least 1 AL biomarker in main analyses, and participants with data on all 8 AL biomarkers in supplemental analyses. | In response to a reviewer comment during peer review, we removed the original main AL analyses on participants with at least 1 biomarker because this approach was not consistent with AL theory. Instead, we moved the original supplemental analyses on the subsample of participants with data on all 8 AL biomarkers to the main text. | | This deviation has minimal impact on reader interpretation of our results, as the significant supplemental results on the aforementioned subsample were the original focal point of the discussion, and this analysis more closely aligns with AL theory. | |
|  | Reason | Peer review |  |  |  |  |  |
|  | Timing | After results known |  |  |  |  |  |
| 8 | Type | Study Design | We originally planned to use full information maximum likelihood (FIML) to address missing data. | We realized that the way our analyses were coded did not achieve FIML; rather, they were essentially conducted using listwise deletion. We corrected this language in the Methods section. | | This deviation has a moderate impact on reader interpretation of our results, as the original data analysis plan was written to include as much data as was reasonable and feasible using FIML. | |
|  | Reason | Typo/Error |  |  |  |  |  |
|  | Timing | After results known |  |  |  |  |  |
| **Unregistered Steps** | | | | | | | |
| # | Details | | Original Wording or Plan | | Unregistered Step Description | | Reader Impact |
| 1 | Type | Inclusion/  exclusion criteria | We planned to include participants missing economic hardship data at one or more waves of measurement if they were not missing data on more than half the waves. | | We included participants in analyses if they had data on at least one of the two measures of economic hardship (income to needs and economic security) for at least half the waves of measurement. These two measures were used to form the composite score of adolescent economic hardship, so we decided to require data on at least one of them for at least half the waves to ensure a sufficiently large sample size for our main analyses. We did not preregister our inclusion criteria with this level of specificity. | | This step introduces researcher degrees of freedom; however, the risk of bias is low given that we made this decision before conducting the analyses. This step has a small impact on readers’ interpretations of our results, because it may lead to misunderstanding of the level of strictness of our inclusion and exclusion criteria. |
|  | Timing | After data access |  |  |  |  |  |
| 2 | Type | Analysis | We planned to test whether economic hardship and perceived discrimination in adolescence predict an increase in depression from age 10 to 26 by controlling for age 10 depression scores. | | We used symptom counts from the Major Depressive Disorder module of the DISC-IV as the measure of age 10 depression. We did not specify the age 10 depression measure in the preregistration. | | This unregistered step has a small impact on readers’ interpretations of our results. It may lead to a misunderstanding that the age 10 depression measure was a similar composite variable to the measure constructed for main analyses of age 26 depression. The risk of bias from this step is low, because we decided on this measure before conducting the analysis. |
|  | Timing | After data access |  |  |  |  |  |
| 3 | Type | Data Preparation | Financial strain was assessed by asking participants’ mothers at every other year of data collection to report on economic stressors using three scales (Conger et al., 1991, 2002).  We planned to average measures of financial strain and income-to-needs ratios within each wave to create an economic hardship variable. | | We had relabeled the financial strain variable “economic pressure” to be consistent with prior publications on this dataset.  However, when reviewing the psychometrics for the composite variable within each wave, we realized that these two variables were inversely correlated because they were on opposite scales. The “economic pressure” scales were originally coded such that higher scores corresponded to higher levels of hardship, but for income-to-needs ratios, lower values corresponded to higher hardship.  To correct this, we only reverse-coded the Can’t Make Ends Meet subscale of the “economic pressure” composite variable and relabeled that composite variable “economic stability.” Then, we recalculated the economic hardship measure and multiplied those composite scores by -1 such that higher scores reflected greater economic hardship. | | This unregistered step has a minor impact on readers’ interpretations of our results, because the economic hardship measure was originally described such that higher scores would reflect greater hardship. This step introduces potential bias in that we discovered that results were originally obtained with an invalid economic hardship measure. Therefore, we needed to reanalyze the economic hardship models with the corrected measure. |
|  | Timing | After results known |  |  |  |  |  |
| 4 | Type | Analysis | The original manuscript included participants with data on at least 1 AL biomarker in main analyses, and participants with data on all 8 AL biomarkers in supplemental analyses. | | After deciding on Deviation 7 described above, we added a supplemental analysis on participants with at least 4 AL biomarkers to balance restriction of inclusion criteria in these analyses. These supplemental analyses by necessity require at least 1 blood biomarker in the AL index. | | This unregistered step has a small impact on reader interpretation of our results. It introduces potential bias because we ran analyses after knowing the original set of results; however, it offers a more balanced approach that maintains consistency with AL theory. |
|  | Timing | After results known |  |  |  |  |  |

*Note*. AL: Allostatic load. DIS-5: Diagnostic Interview Schedule for DSM-5. MASQ: Mood and Anxiety Symptom Questionnaire. DISC-IV: Diagnostic Interview Schedule for Children Version IV. The template and guidance for this table were obtained from Willroth and Atherton (2024).

Table S2

Internal Consistency of Adversity Measures (Cronbach’s Alphas) at Each Assessment Wave

| Wave | Unmet Material Needs | Financial Cutbacks | Can’t Make Ends Meet | Economic Security | Economic Hardship | Perceived Discrimination |
| --- | --- | --- | --- | --- | --- | --- |
| Wave 1 (mean age = 10.87) | .88 | .86 | .73 | .67 | .60 | .58 |
| Wave 2 (mean age = 11.84) | N/A | N/A | N/A | N/A | N/A | .53 |
| Wave 3 (mean age = 12.81) | .84 | .78 | .72 | .66 | .63 | .61 |
| Wave 4 (mean age = 13.80) | N/A | N/A | N/A | N/A | N/A | .74 |
| Wave 5 (mean age = 14.76) | .88 | .69 | .73 | .69 | .65 | .75 |
| Wave 6 (mean age = 15.80) | N/A | N/A | N/A | N/A | N/A | .54 |
| Wave 7 (mean age = 16.80) | .91 | .72 | .76 | .65 | .65 | .59 |
| Wave 8 (mean age = 17.73) | N/A | N/A | N/A | N/A | N/A | .48 |
| Wave 10 (mean age = 19.86) | .92 | .71 | .78 | .70 | .66 | .28 |
| Wave 13 (mean age = 26.13) | .90 | .66 | .75 | .65 | .50 | .91 |

Note: N/A = Not Applicable. The Economic Security score was calculated by summing scores from the Unmet Material Needs, Financial Cutbacks, and Can’t Make Ends Meet scales. Economic Security data were collected every other assessment wave through mother self-report; data on these variables were not obtained at waves 2, 4, 6, or 8. Data were not collected on Economic Security or Perceived Discrimination at wave 9. The Economic Hardship variable is the mean of standardized economic hardship measures (i.e., the Economic Security self-report measures and income-to-needs ratio) multiplied by -1 such that higher values correspond to greater hardship.

Table S3

Descriptive Statistics of Allostatic Load Biomarkers

| Variable (units) | *M* | *SD* | Min | Max |
| --- | --- | --- | --- | --- |
| BMI (kg/m^2^) | 30.55 | 6.47 | 17.58 | 47.61 |
| Waist-to-hip ratio (inches) | 0.88 | 0.08 | 0.68 | 1.17 |
| SBP (mm Hg) | 121.39 | 13.91 | 90.50 | 167.00 |
| DBP (mm Hg) | 78.92 | 9.66 | 55 | 121.50 |
| C-reactive protein (mg/L) | 3.60 | 4.00 | 0.20 | 17.70 |
| Total Cholesterol (mg/dL) | 214.41 | 52.85 | 115.00 | 440.00 |
| HDL Cholesterol (mg/dL) | 51.02 | 15.97 | 7.00 | 127.00 |
| Hemoglobin A1c (%) | 0.05 | 0.008 | 0.04 | 0.12 |
| Triglycerides (mg/dL) | 172.17 | 99.48 | 41.00 | 566.00 |

Note: BMI = Body mass index. SBP = Systolic blood pressure. DBP = Diastolic blood pressure. Blood pressure readings were calculated by computing the average of the second and third measurements. Higher levels of HDL cholesterol indicate better health, so risk quartiles for this biomarker were reversed before calculating the sum allostatic load score. Hemoglobin A1c percentages were converted to numbers for analyses and descriptive statistics (i.e., 5% = 0.05).

Table S4

Sample Sizes for Main Analyses

| Model | *N* |
| --- | --- |
| Adolescent discrimination predicting young adult AL | 212 |
| Adolescent discrimination predicting young adult depression | 484 |
| Adolescent economic hardship predicting young adult AL | 210 |
| Adolescent economic hardship predicting young adult depression | 481 |
| Association between young adult AL and depression | 215 |
| Adolescent discrimination X young adult depression predicting AL | 210 |
| Adolescent economic hardship X young adult depression predicting AL | 208 |
| Resilience to adolescent discrimination predicting young adult AL | 210 |
| Resilience to adolescent economic hardship predicting young adult AL | 208 |

Note: AL = allostatic load.

**Supplementary Figures**

Figure S1.

*Greater perceived discrimination in adolescence predicted higher levels of depression at age 26.*


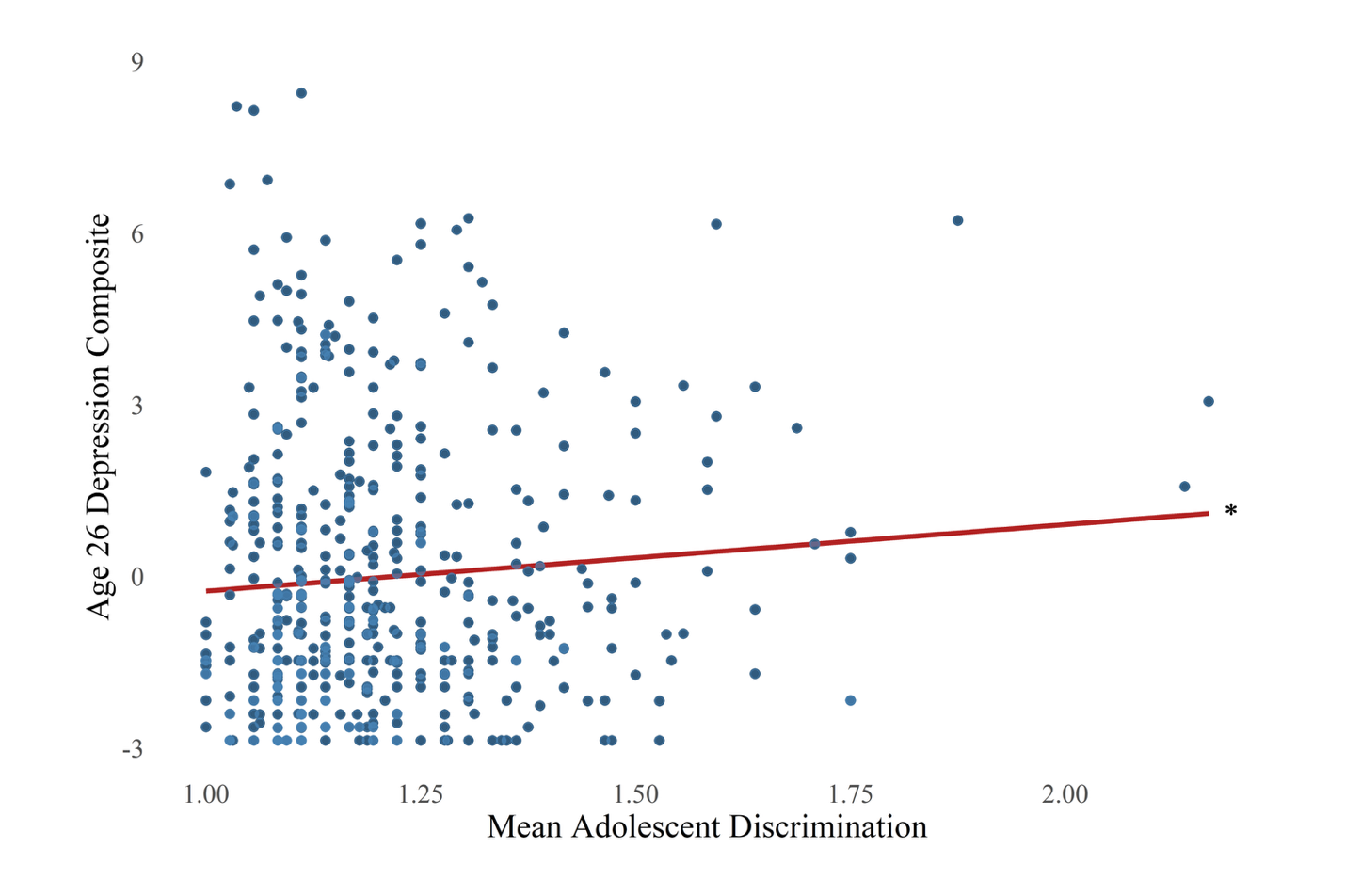


*Note*. * *p* < .05 (slope is significantly different from zero).

**Supplementary References**

Bey, G.S., Jesdale, B.M., Ulbricht, C.M., Mick, E.O., Person, S.D., 2018. Allostatic Load Biomarker Associations with Depressive Symptoms Vary among US Black and White Women and Men. Healthcare 6, 105. https://doi.org/10.3390/healthcare6030105

Cohen, J., 1988. Statistical Power Analysis for the Behavioral Sciences, 2nd ed. Routledge, New York. https://doi.org/10.4324/9780203771587

Conger, R.D., Lorenz, F.O., Elder, G.H., Melby, J.N., Simons, R.L., Conger, K.J., 1991. A Process Model of Family Economic Pressure and Early Adolescent Alcohol Use. The Journal of Early Adolescence 11, 430–449. https://doi.org/10.1177/0272431691114003

Conger, R.D., Wallace, L.E., Sun, Y., Simons, R.L., McLoyd, V.C., Brody, G.H., 2002. Economic pressure in African American families: A replication and extension of the family stress model. Developmental Psychology 38, 179–193. https://doi.org/10.1037/0012-1649.38.2.179

da Silva-Sauer, L., Basso Garcia, R., Pereira da Silva, T.M., Barbosa de Melo, C., Fernández-Calvo, B., 2021. Relationship between psychological resilience, perceived stress, depression, and physical health in community-dwelling older adults. Psychology & Neuroscience 14, 132–144. https://doi.org/10.1037/pne0000254

Evans, G.W., De France, K., 2022. Childhood Poverty and Psychological Wellbeing: The Mediating Role of Cumulative Risk Exposure. Dev Psychopathol 34, 911–921. https://doi.org/10.1017/S0954579420001947

Faul, F., Erdfelder, E., Lang, A.-G., Buchner, A., 2007. G*Power 3: A flexible statistical power analysis program for the social, behavioral, and biomedical sciences. Behavior Research Methods 39, 175–191. https://doi.org/10.3758/BF03193146

Flores, E., Tschann, J.M., Dimas, J.M., Bachen, E.A., Pasch, L.A., Groat, C.L. de, 2008. Perceived Discrimination, Perceived Stress, and Mental and Physical Health Among Mexican-Origin Adults. Hispanic Journal of Behavioral Sciences. https://doi.org/10.1177/0739986308323056

Gale, C.R., Batty, G.D., Cooper, S.-A., Deary, I.J., Der, G., McEwen, B.S., Cavanagh, J., 2015. Reaction Time in Adolescence, Cumulative Allostatic Load, and Symptoms of Anxiety and Depression in Adulthood: The West of Scotland Twenty-07 Study. Psychosomatic Medicine 77, 493. https://doi.org/10.1097/PSY.0000000000000189

Johnston, K.E., Delgado, M.Y., 2004. Mexican American adolescents’ experiences with ethnic discrimination.

Linnemann, P., Berger, K., Teismann, H., 2022. Associations Between Outcome Resilience and Sociodemographic Factors, Childhood Trauma, Personality Dimensions and Self-Rated Health in Middle-Aged Adults. Int.J. Behav. Med. 29, 796–806. https://doi.org/10.1007/s12529-022-10061-1

Lorant, V., Deliège, D., Eaton, W., Robert, A., Philippot, P., Ansseau, M., 2003. Socioeconomic Inequalities in Depression: A Meta-Analysis. American Journal of Epidemiology 157, 98–112. https://doi.org/10.1093/aje/kwf182

Richardson, L.J., Goodwin, A.N., Hummer, R.A., 2021. Social status differences in allostatic load among young adults in the United States. SSM - Population Health 15, 100771. https://doi.org/10.1016/j.ssmph.2021.100771

Shaffer, D., Fisher, P., Lucas, C.P., Dulcan, M.K., Schwab-stone, M.E., 2000. NIMH Diagnostic Interview Schedule for Children Version IV (NIMH DISC-IV): Description, Differences From Previous Versions, and Reliability of Some Common Diagnoses. Journal of the American Academy of Child & Adolescent Psychiatry 39, 28–38. https://doi.org/10.1097/00004583-200001000-00014

Upchurch, D.M., Stein, J., Greendale, G.A., Chyu, L., Tseng, C.-H., Huang, M.-H., Lewis, T.T., Kravitz, H.M., Seeman, T., 2015. A Longitudinal Investigation of Race, Socioeconomic Status, and Psychosocial Mediators of Allostatic Load in Midlife Women: Findings From the Study of Women’s Health Across the Nation. Psychosomatic Medicine 77, 402. https://doi.org/10.1097/PSY.0000000000000175

Weissman, D.G., Conger, R.D., Robins, R.W., Hastings, P.D., Guyer, A.E., 2018. Income change alters default mode network connectivity for adolescents in poverty. Developmental Cognitive Neuroscience 30, 93–99. https://doi.org/10.1016/j.dcn.2018.01.008

Weissman, M.M., Bland, R., Joyce, P.R., Newman, S., Wells, J.E., Wittchen, H.-U., 1993. Sex differences in rates of depression: cross-national perspectives. Journal of Affective Disorders, Special Issue Toward a New Psychobiology of Depression in Women 29, 77–84. https://doi.org/10.1016/0165-0327(93)90025-F

Willroth, E.C., Atherton, O.E., 2024. Best Laid Plans: A Guide to Reporting Preregistration Deviations. Advances in Methods and Practices in Psychological Science 7, 25152459231213802. https://doi.org/10.1177/25152459231213802
