# Supplementary material for: Associations between adolescent adversity and young adult depression symptoms and allostatic load in Mexican-origin individuals
Source: Psychoneuroendocrinology. Author manuscript; Available in PMC 2026 Jun 24. (PMC13293614; doi:10.1016/j.psyneuen.2026.107832)
Supplement: 2 [file NIHMS2180036-supplement-2.docx]

| Table S1 | | | | | | | |
| --- | --- | --- | --- | --- | --- | --- | --- |
| *Preregistration Deviations* | | | | | | | |
| **Deviations** | | | | | | | |
| # | Details | | Original Wording or Plan | Deviation Description | | Reader Impact | |
| 1 | Type | Variables | We planned to include total hemoglobin levels obtained from blood samples as one of the 9 biomarkers in our AL measure. | We did not obtain total hemoglobin. Total hemoglobin tests for anemia; however, we did not have hypotheses about anemia, and this biomarker would require a second blood draw. Therefore, we had decided not to measure it. This biomarker was included in the preregistration in error and the number of biomarkers in the AL measure was 8 rather than 9. | | This deviation has a minor impact on readers’ interpretations of our results. It resulted from an oversight when writing the preregistration and was corrected prior to data analysis, when preparing the data. Because we did not already know the results when we made this correction, the risk of bias is low. The small difference in interpretation of results is that our AL measure does not reflect anemia. | |
|  | Reason | Miscommunication |  |  |  |  |  |
|  | Timing | After data access |  |  |  |  |  |
| 2 | Type | Variables | The Financial Cutbacks scale consisted of **7 items** asking about their adjustments to financial need (1 = yes, 0 = no) during the past 3 months. | This scale has 9 items. The number of items described in the preregistration was an error. | | This deviation has minimal impact on readers’ interpretations of the results. It may lead to misunderstandings of the extent to which our economic hardship composite score reflected financial cutbacks. The risk of bias is low because we recognized this error before analyzing the data. | |
|  | Reason | Typo/Error |  |  |  |  |  |
|  | Timing | After data access |  |  |  |  |  |
| 3 | Type | Data Preparation | The Financial Cutbacks scale consisted of 7 items asking about their adjustments to financial need (**1 = yes, 0 = no**) during the past 3 months. | This scale was coded in the dataset as 1 = yes, 2 = no. | | This deviation has minimal impact on readers’ interpretations of our findings. We miscommunicated the way this variable was coded in data entry in the preregistration. | |
|  | Reason | Typo/Error |  |  |  |  |  |
|  | Timing | After data access |  |  |  |  |  |
| 4 | Type | Data Preparation | To quantify depression symptoms, we will create a single composite variable by standardizing the measures of symptom counts from the major depression module of the DIS-5 as well as the Anhedonic Depression and General Distress subscales of the MASQ and then summing the standardized measures. | We averaged the MASQ subscales rather than computing symptom counts to be consistent with prior publications on this dataset. | | This deviation has minimal impact on readers’ interpretations of our findings. The MASQ subscale scores should have been described as averages in the preregistration. | |
|  | Reason | Typo/Error |  |  |  |  |  |
|  | Timing | After results known |  |  |  |  |  |
| 5 | Type | Data Preparation | For analyses, we will use sum scores from the Personal  Experiences with Prejudice and Discrimination subscale that asks about adolescents’ personal experiences with discrimination. | We averaged scores from the Personal Experiences with Prejudice and Discrimination subscale within each wave to be consistent with prior publications on this dataset. | | This deviation has minimal impact on readers’ interpretations of our findings. These subscale scores should have been described as averages in the preregistration. | |
|  | Reason | Typo/Error |  |  |  |  |  |
|  | Timing | After results known |  |  |  |  |  |
| 6 | Type | Other (Please Explain) | For research questions where the same model is tested but with a different adversity measure, we wrote that we would conduct a false discovery rate correction to control for multiple testing. | We intended to write that we would conduct the more conservative correction for family-wise error rate using the Bonferroni procedure. A false discovery rate correction would be too liberal to use with our analyses because we only conducted two statistical tests for each model, corresponding to the two forms of adversity. | | This deviation has moderate impact on readers’ interpretations of our findings, as they may expect our correction procedure to be less conservative. We specified the accurate correction procedure in the method section and clarified in the discussion that our results did not survive correction for multiple comparisons. | |
|  | Reason | Typo/Error |  |  |  |  |  |
|  | Timing | After results known |  |  |  |  |  |
| 7 | Type | Analysis | The original manuscript included participants with data on at least 1 AL biomarker in main analyses, and participants with data on all 8 AL biomarkers in supplemental analyses. | In response to a reviewer comment during peer review, we removed the original main AL analyses on participants with at least 1 biomarker because this approach was not consistent with AL theory. Instead, we moved the original supplemental analyses on the subsample of participants with data on all 8 AL biomarkers to the main text. | | This deviation has minimal impact on reader interpretation of our results, as the significant supplemental results on the aforementioned subsample were the original focal point of the discussion, and this analysis more closely aligns with AL theory. | |
|  | Reason | Peer review |  |  |  |  |  |
|  | Timing | After results known |  |  |  |  |  |
| 8 | Type | Study Design | We originally planned to use full information maximum likelihood (FIML) to address missing data. | We realized that the way our analyses were coded did not achieve FIML; rather, they were essentially conducted using listwise deletion. We corrected this language in the Methods section. | | This deviation has a moderate impact on reader interpretation of our results, as the original data analysis plan was written to include as much data as was reasonable and feasible using FIML. | |
|  | Reason | Typo/Error |  |  |  |  |  |
|  | Timing | After results known |  |  |  |  |  |
| **Unregistered Steps** | | | | | | | |
| # | Details | | Original Wording or Plan | | Unregistered Step Description | | Reader Impact |
| 1 | Type | Inclusion/  exclusion criteria | We planned to include participants missing economic hardship data at one or more waves of measurement if they were not missing data on more than half the waves. | | We included participants in analyses if they had data on at least one of the two measures of economic hardship (income to needs and economic security) for at least half the waves of measurement. These two measures were used to form the composite score of adolescent economic hardship, so we decided to require data on at least one of them for at least half the waves to ensure a sufficiently large sample size for our main analyses. We did not preregister our inclusion criteria with this level of specificity. | | This step introduces researcher degrees of freedom; however, the risk of bias is low given that we made this decision before conducting the analyses. This step has a small impact on readers’ interpretations of our results, because it may lead to misunderstanding of the level of strictness of our inclusion and exclusion criteria. |
|  | Timing | After data access |  |  |  |  |  |
| 2 | Type | Analysis | We planned to test whether economic hardship and perceived discrimination in adolescence predict an increase in depression from age 10 to 26 by controlling for age 10 depression scores. | | We used symptom counts from the Major Depressive Disorder module of the DISC-IV as the measure of age 10 depression. We did not specify the age 10 depression measure in the preregistration. | | This unregistered step has a small impact on readers’ interpretations of our results. It may lead to a misunderstanding that the age 10 depression measure was a similar composite variable to the measure constructed for main analyses of age 26 depression. The risk of bias from this step is low, because we decided on this measure before conducting the analysis. |
|  | Timing | After data access |  |  |  |  |  |
| 3 | Type | Data Preparation | Financial strain was assessed by asking participants’ mothers at every other year of data collection to report on economic stressors using three scales (Conger et al., 1991, 2002).  We planned to average measures of financial strain and income-to-needs ratios within each wave to create an economic hardship variable. | | We had relabeled the financial strain variable “economic pressure” to be consistent with prior publications on this dataset.  However, when reviewing the psychometrics for the composite variable within each wave, we realized that these two variables were inversely correlated because they were on opposite scales. The “economic pressure” scales were originally coded such that higher scores corresponded to higher levels of hardship, but for income-to-needs ratios, lower values corresponded to higher hardship.  To correct this, we only reverse-coded the Can’t Make Ends Meet subscale of the “economic pressure” composite variable and relabeled that composite variable “economic stability.” Then, we recalculated the economic hardship measure and multiplied those composite scores by -1 such that higher scores reflected greater economic hardship. | | This unregistered step has a minor impact on readers’ interpretations of our results, because the economic hardship measure was originally described such that higher scores would reflect greater hardship. This step introduces potential bias in that we discovered that results were originally obtained with an invalid economic hardship measure. Therefore, we needed to reanalyze the economic hardship models with the corrected measure. |
|  | Timing | After results known |  |  |  |  |  |
| 4 | Type | Analysis | The original manuscript included participants with data on at least 1 AL biomarker in main analyses, and participants with data on all 8 AL biomarkers in supplemental analyses. | | After deciding on Deviation 7 described above, we added a supplemental analysis on participants with at least 4 AL biomarkers to balance restriction of inclusion criteria in these analyses. These supplemental analyses by necessity require at least 1 blood biomarker in the AL index. | | This unregistered step has a small impact on reader interpretation of our results. It introduces potential bias because we ran analyses after knowing the original set of results; however, it offers a more balanced approach that maintains consistency with AL theory. |
|  | Timing | After results known |  |  |  |  |  |

*Note*. AL: Allostatic load. DIS-5: Diagnostic Interview Schedule for DSM-5. MASQ: Mood and Anxiety Symptom Questionnaire. DISC-IV: Diagnostic Interview Schedule for Children Version IV. The template and guidance for this table were obtained from Willroth and Atherton (2024).
