# Supplementary material for: Associations between adolescent adversity and young adult depression symptoms and allostatic load in Mexican-origin individuals
Source: Psychoneuroendocrinology. Author manuscript; Available in PMC 2026 Jun 24. (PMC13293614; doi:10.1016/j.psyneuen.2026.107832)
Supplement: 3 [file NIHMS2180036-supplement-3.docx]

Table S2

Internal Consistency of Adversity Measures (Cronbach’s Alphas) at Each Assessment Wave

| Wave | Unmet Material Needs | Financial Cutbacks | Can’t Make Ends Meet | Economic Security | Economic Hardship | Perceived Discrimination |
| --- | --- | --- | --- | --- | --- | --- |
| Wave 1 (mean age = 10.87) | .88 | .86 | .73 | .67 | .60 | .58 |
| Wave 2 (mean age = 11.84) | N/A | N/A | N/A | N/A | N/A | .53 |
| Wave 3 (mean age = 12.81) | .84 | .78 | .72 | .66 | .63 | .61 |
| Wave 4 (mean age = 13.80) | N/A | N/A | N/A | N/A | N/A | .74 |
| Wave 5 (mean age = 14.76) | .88 | .69 | .73 | .69 | .65 | .75 |
| Wave 6 (mean age = 15.80) | N/A | N/A | N/A | N/A | N/A | .54 |
| Wave 7 (mean age = 16.80) | .91 | .72 | .76 | .65 | .65 | .59 |
| Wave 8 (mean age = 17.73) | N/A | N/A | N/A | N/A | N/A | .48 |
| Wave 10 (mean age = 19.86) | .92 | .71 | .78 | .70 | .66 | .28 |
| Wave 13 (mean age = 26.13) | .90 | .66 | .75 | .65 | .50 | .91 |

Note: N/A = Not Applicable. The Economic Security score was calculated by summing scores from the Unmet Material Needs, Financial Cutbacks, and Can’t Make Ends Meet scales. Economic Security data were collected every other assessment wave through mother self-report; data on these variables were not obtained at waves 2, 4, 6, or 8. Data were not collected on Economic Security or Perceived Discrimination at wave 9. The Economic Hardship variable is the mean of standardized economic hardship measures (i.e., the Economic Security self-report measures and income-to-needs ratio) multiplied by -1 such that higher values correspond to greater hardship.
