# Supplementary material for: Associations between adolescent adversity and young adult depression symptoms and allostatic load in Mexican-origin individuals
Source: Psychoneuroendocrinology. Author manuscript; Available in PMC 2026 Jun 24. (PMC13293614; doi:10.1016/j.psyneuen.2026.107832)
Supplement: 4 [file NIHMS2180036-supplement-4.docx]

Table S3

Descriptive Statistics of Allostatic Load Biomarkers

| Variable (units) | *M* | *SD* | Min | Max |
| --- | --- | --- | --- | --- |
| BMI (kg/m^2^) | 30.55 | 6.47 | 17.58 | 47.61 |
| Waist-to-hip ratio (inches) | 0.88 | 0.08 | 0.68 | 1.17 |
| SBP (mm Hg) | 121.39 | 13.91 | 90.50 | 167.00 |
| DBP (mm Hg) | 78.92 | 9.66 | 55 | 121.50 |
| C-reactive protein (mg/L) | 3.60 | 4.00 | 0.20 | 17.70 |
| Total Cholesterol (mg/dL) | 214.41 | 52.85 | 115.00 | 440.00 |
| HDL Cholesterol (mg/dL) | 51.02 | 15.97 | 7.00 | 127.00 |
| Hemoglobin A1c (%) | 0.05 | 0.008 | 0.04 | 0.12 |
| Triglycerides (mg/dL) | 172.17 | 99.48 | 41.00 | 566.00 |

Note: BMI = Body mass index. SBP = Systolic blood pressure. DBP = Diastolic blood pressure. Blood pressure readings were calculated by computing the average of the second and third measurements. Higher levels of HDL cholesterol indicate better health, so risk quartiles for this biomarker were reversed before calculating the sum allostatic load score. Hemoglobin A1c percentages were converted to numbers for analyses and descriptive statistics (i.e., 5% = 0.05).
