# Supplementary material for: Associations between adolescent adversity and young adult depression symptoms and allostatic load in Mexican-origin individuals
Source: Psychoneuroendocrinology. Author manuscript; Available in PMC 2026 Jun 24. (PMC13293614; doi:10.1016/j.psyneuen.2026.107832)
Supplement: 5 [file NIHMS2180036-supplement-5.docx]

Table S4

Sample Sizes for Main Analyses

| Model | *N* |
| --- | --- |
| Adolescent discrimination predicting young adult AL | 212 |
| Adolescent discrimination predicting young adult depression | 484 |
| Adolescent economic hardship predicting young adult AL | 210 |
| Adolescent economic hardship predicting young adult depression | 481 |
| Association between young adult AL and depression | 215 |
| Adolescent discrimination X young adult depression predicting AL | 210 |
| Adolescent economic hardship X young adult depression predicting AL | 208 |
| Resilience to adolescent discrimination predicting young adult AL | 210 |
| Resilience to adolescent economic hardship predicting young adult AL | 208 |

Note: AL = allostatic load.
